# Lifestyle-associated gut microbiota signatures in colorectal cancer patients: a pilot case–control study

**DOI:** 10.7717/peerj.21829

**Published:** 2026-10-27

**Authors:** Sara M. ElAdawy, Hend Talkhan, Khalid Orayj, Gehan F. Balata, Marwa Saeed, Ahmed A. Abdelkader, Hany N. Azzam, Noha A. Abdelrazek, Inas Abdou Mahmoud, Mostafa ElHaddad, Sama M. Momen, Maram Hamada, Mayar Hamada, Alaa O. Elyousefy, Kholoud A. Hashem, Mona A. Alshikhari, Ghena M. Deeb, Sohair M. Elgohary, Masa M. Albakri, Amany Mohamed, Toka Arshad, Nadra S. El-Sayed, Rahma S. Etman, Mohaned M. Nawar, Esraa E. Sobhy

**Affiliations:** 1 Integrative Health Center, Heliopolis University, Cairo, Egypt; 2 Department of Pharmacy Practice, Faculty of Pharmacy, Heliopolis University, Cairo, Egypt; 3 Department of Clinical Pharmacy, College of Pharmacy, King Khalid University, Abha, Saudi Arabia; 4 Department of Clinical Pharmacy, Kasr Al Aini Hospital, Cairo, Egypt; 5 Department of Clinical Oncology and Nuclear Medicine, Kasr Al Aini Hospital, Cairo, Egypt; 6 Faculty of Pharmacy, Heliopolis University, Cairo, Egypt; 7 Department of Microbiology and Immunology, Faculty of Pharmacy, Delta University for Science and Technology, Gamasa, Egypt

**Keywords:** Colorectal cancer, Microbial diversity, Diet, Physical activity, Dysbiosis, Egypt, Lifestyle, Behavior

## Abstract

**Background and Objectives:**

Colorectal cancer (CRC) is the third most common cancer in men and the second in women, accounting for 9.4% of cancer-related deaths worldwide in 2020. Increasing evidence highlights the role of gut microbiota in CRC pathogenesis, prognosis, and therapeutic response. Data from Middle Eastern and North African populations remains scarce. This study aimed to analyze gut microbiota profiles in Egyptian patients with CRC and to explore their associations with dietary and lifestyle factors.

**Material and Methods:**

A pilot case-control study was conducted, involving patients with CRC and healthy controls. Gut microbiota composition was assessed using 16S rRNA gene sequencing, with analysis of microbial diversity and differential taxonomic abundance at the family and genus levels. Dietary patterns, physical activity, and demographic variables were evaluated for their associations with microbial profiles.

**Results:**

CRC patients exhibited greater microbial heterogeneity, with higher Bray–Curtis distances compared to controls. Several taxa, such as *Coprococcus_2*, *Ruminococcus*, Blautia, and Dorea, were abundant in controls and adjacent normal tissues, whereas Lachnospiraceae UCG-010 was increased in CRC samples. Spicy food intake was linked to enrichment of *Prevotellaceae* and *Christensenellaceae R-7*, while processed meat consumption was associated with reduced abundance of Fusicatenibacter. Regular physical activity was associated with increased *Akkermansia* abundance among CRC patients. Additionally, sex-specific microbial patterns were observed, with *Collinsella*, *Eggerthella*, and *Prevotella*_*9* more abundant in males.

**Conclusions:**

This pilot study identifies clinically relevant, exploratory associations between lifestyle factors and CRC-related microbial signatures in an understudied population. All findings should be considered hypothesis-generating, given the small sample size and cross-sectional design. These results highlight the potential role of modifiable behaviors in microbiome-informed risk stratification. Larger, multi-center studies incorporating multi-omics approaches are warranted to confirm and extend these preliminary observations.

## Introduction

Colorectal cancer (CRC) is the third most commonly diagnosed cancer in men and the second in women worldwide, and accounted for about 9.4% of all cancer-related deaths in 2020 (Park et al., 2021). The majority of cases occur in individuals aged 50 years and older (Park et al., 2021). In Egypt, CRC is estimated to be the seventh most common cancer diagnosis (Sung et al., 2021; Gado et al., 2016), with a reported prevalence of approximately 9.8 cases per 100,000 population (GBD 2019 Colorectal Cancer Collaborators, 2022). In contrast to Western countries, where CRC incidence rates are among the highest globally (*e.g.*, approximately 35–40 per 100,000 in the United States), Egypt and other North African countries report considerably lower rates, although incidence is rising, particularly among younger adults. Differences in dietary patterns, environmental exposures, lifestyle factors, and gut microbiota composition may contribute to these epidemiological differences and warrant further investigation (Hassan, Khalaf & Elias, 2021).

Billions of dollars are spent on healthcare systems worldwide due to CRC, with costs varying by disease stage, treatment type, and geographic location (Orangio, 2018; Jafari et al., 2021). In Egypt, the mean direct medical cost for CRC is $610 per patient per year, representing approximately 80% of total expenditures; chemotherapy and surgery account for 4.7% and radiotherapy for 1.7% of expenses. Costs are substantially higher in the metastatic phase compared with the non-metastatic phase (stages I–III) (Abotaleb, 2018).

Increasing evidence indicates that CRC is influenced by environmental factors, particularly through interactions with the gut microbiota, opening new possibilities for early detection, prognosis, and treatment. Bacterial taxa, including *Fusobacterium, Enterococcus*, and *Prevotella,* have been linked to mucosal gene expression patterns and may have a role in the development of CRC mucosa. The abundance of these bacteria has been reported to be altered in CRC patients. Additionally, studies have indicated that people infected with *Salmonella enterica* had a higher prevalence of CRC compared with individuals with normal colonic mucosa (Repass, Maherali & Owen, 2016).

Multiple models and theories have been proposed to explain the role of the microbiome in CRC, including the alpha-bug hypothesis, which postulates that specific bacterial species may induce cancer and alter the bacterial community in a way that promotes the spread of cancer (Maddocks et al., 2009). Another hypothesis is the driver-passenger model, which distinguishes between driver and passenger bacteria in the microbiota and explains variations in study results (Tjalsma et al., 2012; Yu & Fang, 2015).

Diet and lifestyle significantly influence gut microbiota composition and metabolic activity (GBD 2019 Colorectal Cancer Collaborators, 2022). Microbial metabolites, particularly bile acids and short-chain fatty acids, play a key role in the interaction between microbes and the host, and can exert either protective or pro-carcinogenic effects (Baas et al., 2024; Singhal et al., 2023). Diet-induced dysbiosis can promote inflammation, the formation of genotoxic metabolites, and immune disruption, thereby contributing to colorectal tumorigenesis (Jafari et al., 2021). Recent metagenomic and metabolomic studies have clarified these diet–microbiome–CRC associations, with meta-analyses identifying key CRC-related microbes, such as *Fusobacterium nucleatum*, *Peptostreptococcus stomatis*, *Gemella morbillorum*, *Bacteroides fragilis, Parvimonas spp., Clostridium symbiosum,* and *Solobacterium moorei* (Huycke, Abrams & Moore, 2002). However, most available data originate from Western and East Asian populations, and information from Middle Eastern and North African countries, including Egypt, remains limited.

Therefore, this pilot case-control study aimed to analyze gut microbiota profiles in Egyptian patients with CRC and to explore their associations with dietary and lifestyle factors.

## Materials & Methods

### Study design and setting

A pilot case-control study was conducted at the Department of Oncology and Nuclear Medicine, Kasr Al Aini Hospital, Cairo University, Cairo, Egypt. Recruitment and sample collection were undertaken over six months, with patient follow-up visits of four days per week. The study protocol was approved by the Research Ethics Committees of Heliopolis University (Approval No. HU.REC.H.10-2024) and the Institutional Review Board of Kasr Al Aini Hospital, Cairo University (Approval No. N-404-2024), and was conducted in accordance with the Declaration of Helsinki. Written informed consent was obtained from all participants involved in the study.

### Study population

A total of 15 participants were enrolled and allocated into two groups: (1) patients with newly diagnosed CRC and (2) healthy controls. The CRC diagnosis was confirmed histopathologically, through examination of tissue specimens at the pathology laboratory of Kasr Al Aini Hospital or another accredited pathology laboratory.

### Inclusion and exclusion criteria

Adults aged 40 to 80 years with a newly diagnosed CRC were included. Participants were excluded if they had a previous history of CRC, pregnancy, lactation, abdominal surgery within/up to 6 months before sample collection, antibiotic, corticosteroid, or probiotic use up to three months before sample collection, a history of other types of cancer, diabetes mellitus, or chronic liver or kidney disease. Patients who adhered to a special diet or used enemas/colonoscopy up to one week before sample collection were also excluded.

### Data collection

Demographic and clinical data were obtained through structured interviews and review of electronic medical records. Lifestyle and dietary variables included the consumption of fast food, spicy food, canned fish/tuna, broasted chicken, processed chicken, processed meat, coffee (*e.g.*, caffeinated instant coffee), canned juices, tap water, and physical activity, as well as age and sex.

Dietary intake was evaluated using a structured questionnaire culturally adapted for the Egyptian population from a previously developed modified dietary assessment tool (Nielsen et al., 2023).

The questionnaire was reviewed by a panel of medical experts to ensure linguistic clarity and content validity. Consumption of a food item was considered regular if it occurred at least once per week (≥1 time/week). Nutrient content (carbohydrates, protein, fat, and dietary fiber) was derived using the Egyptian food composition database (National Nutrition Institute, NNI).

All interviews were conducted by well-trained health care professionals (pharmacists and physicians). Consequently, language barriers were minimized (Data S1).

### Stool sampling, DNA extraction, and 16S rRNA sequencing

Fecal samples were collected, and microbial DNA was extracted using the DNeasy PowerSoil DNA Extraction Kit (Qiagen, Hilden, Germany), following the manufacturer’s protocol. DNA concentration was assessed using both the Nanodrop spectrophotometer and Qubit Broad Range (BR) assay, while purity was evaluated *via* Nanodrop A260/280 and A260/230 ratios.

Approximately 30 ng of high-purity DNA per sample was used for amplification targeting the V3–V4 region of the 16S rRNA gene using fusion primers (forward: ACTCCTACGGGAGGCAGCAG) and (reverse: GGACTACHVGGGTWTCTAAT). Amplicons were purified using Agencourt AMPure XP magnetic beads and subsequently eluted in an elution buffer. Following purification, DNA libraries were constructed and quality checked using the Agilent 2100 Bioanalyzer. Sequencing was performed on the DNBSEQ platform (BGI, Shenzhen, China).

### Bioinformatics and microbiome analysis

Raw sequencing data were processed using the DADA2 pipeline (Callahan et al., 2016) in R programming language (version 4.2.3), with key packages including microeco (McMurdie & Holmes, 2013), phyloseq (Liu et al., 2021a), DECIPHER (Wright, 2016), phangorn (Schliep, 2011), and ggplot2 (Ginestet, 2011). Initial preprocessing was performed by the sequencing provider. Primer and adapter sequences were removed using Cutadapt. Reads were quality filtered by truncating reads when the average Phred quality score fell below 20 within a 30-bp sliding window, and reads shorter than 75% of their original length after truncation were discarded. Reads containing ambiguous bases were removed, and low-complexity reads, defined as reads containing 10 consecutive identical bases, were excluded. Paired-end reads were merged using FLASH, requiring a minimum overlap length of 15 bp and a maximum mismatch ratio of 0.1 in the overlapping region.

Further amplicon sequence variant (ASV) processing was performed in R using the DADA2 pipeline. Forward and reverse reads were filtered and trimmed using *filterAndTrim* with trimLeft = 10, truncLen = c(240, 200), maxN = 0, maxEE = c(2, 5), and truncQ = 2. Error models were learned separately for forward and reverse reads using learnErrors, and ASVs were inferred using the dada function. Forward and reverse reads were merged using *mergePairs*, and the ASV table was constructed using *makeSequenceTable*. Chimeric sequences were removed using *removeBimeraDenovo*. The final non-chimeric ASV table was exported for downstream analysis.

A multiple sequence alignment was performed on the ASV sequences, and phylogenetic trees were generated using the neighbor-joining method (Saitou & Nei, 1987), followed by maximum likelihood (ML) optimization under the GTR+G+I model (Felsenstein, 1981). Although a phylogenetic tree was generated from aligned ASV sequences for downstream compatibility, the primary beta-diversity analyses reported in this study were based on Bray–Curtis dissimilarities and did not use UniFrac distances.

Taxonomic assignment was performed using the SILVA reference database [v138] (Quast et al., 2013). To account for differences in sequencing depth between samples, normalization was performed using scaling with ranked subsampling (SRS), a normalization approach designed to generate equal library sizes while minimizing information loss associated with conventional rarefaction. Following normalization, all samples were standardized to an equal sequencing depth of 21,088 reads prior to downstream diversity analyses.

Alpha, beta diversity, and downstream analyses were conducted using normalized count data. Alpha diversity metrics were computed and compared using Wilcoxon rank-sum tests. Within-group microbial heterogeneity was assessed by calculating pairwise Bray–Curtis dissimilarities among samples within each group. The distributions of within-group pairwise distances were compared between CRC patients and controls using Wilcoxon rank-sum testing.

To investigate potential contributors to beta diversity variation, exploratory multifactorial PERMANOVA analyses were initially performed using Bray–Curtis dissimilarity matrices, incorporating disease status together with individual dietary or lifestyle variables. Additional multivariable PERMANOVA analyses were subsequently performed, incorporating disease status together with selected demographic and dietary covariates within a single model. Marginal effects were assessed using permutation testing (999 permutations). Variables demonstrating significant associations in PERMANOVA analyses were further assessed using homogeneity of multivariate dispersion testing (*betadisper*) to evaluate whether observed differences reflected variance heterogeneity rather than differences in centroid location.

Differential abundance analyses for dietary and lifestyle subgroup comparisons were performed using the metagenomeSeq zero-inflated log-normal model at the genus level (Paulson et al., 2013). Because multiple subgroup analyses were conducted across several dietary, lifestyle, and demographic variables, raw *p*-values generated from all subgroup differential abundance analyses were pooled and subjected to false discovery rate (FDR) correction using the Benjamini–Hochberg method to reduce inflation of type I error associated with repeated testing. FDR-adjusted *p-values <0.05* were considered statistically significant. Given the limited cohort size and genus-level aggregation, differential abundance findings should be considered exploratory and hypothesis-generating.

### Statistical data analysis

Categorical variables were presented as frequencies and percentages, while continuous variables were expressed as mean ± standard deviation (SD) or median with interquartile range (IQR) according to distribution. The characteristics of the CRC patients and control group were compared using Fisher’s exact test for categorical variables and the Independent Samples *t*-test or Mann–Whitney U test for continuous variables. Statistical analyses were performed using Statistical Package for the Social Sciences (SPSS) version 28 (IBM Corp., Armonk, NY, USA), and a *p*-value < 0.05 was considered statistically significant. Potential overlap among dietary variables was assessed using pairwise association analyses, including Cramer’s V and Fisher’s exact tests, with detailed results provided in Data S2.

## Results

### Characteristics of the participants

A total of 15 participants were included, comprising 7 patients with CRC and 8 healthy controls (Table 1). Both groups were predominantly female (57.1% in the CRC group *vs.* 62.5% in controls, *p* = 1.0). This predominantly female composition likely reflects the demographic profile of patients attending the oncology clinic at Kasr Al Aini Hospital and higher willingness to participate among female volunteers; sex distribution differences between Egyptian and Western CRC populations are acknowledged as a study limitation. Patients with CRC were significantly younger than controls (mean age 42.6 ± 12.4 years in cases *vs.* 56.9 ± 8.0 years in controls, *p* = 0.02). This difference likely reflects the recruitment setting—a tertiary oncology center where younger patients with more aggressive or early-onset disease may be over-represented—and the exclusion of older individuals with multiple comorbidities. The age imbalance is acknowledged as a key limitation, given that gut microbiota composition is significantly influenced by age.

**Table 1:** Characteristics of colorectal cancer patients and healthy controls.

Characteristics	CRC cases (*n* = 7)	Controls (*n* = 8)	*P* value
1-DEMOGRAPHIC AND CLINICAL			
Gender			1.0^a^
Male	3 (42.9)	3 (37.5)	
Female	4 (57.1)	5 (62.5)	
Age (years), mean ± SD	42.6 ± 12.4	56.9 ± 8.0	**0.02** ^*^ ^b^
BMI (kg/m^2^), mean ± SD	25.0 ± 5.9	26.9 ± 2.4	0.43^b^
Normal bowel habits			**0.01** ^*^ ^a^
No	5 (71.4)	0 (0)	
Yes	2 (28.6)	8 (100.0)	
Tumor location			N/A
Ascending colon	1 (14.3)	N/A	
Descending colon	4 (57.1)		
Sigmoid colon	1 (14.3)		
Rectum	1 (14.3)		
Tumor stage			N/A
I	1 (14.3)	N/A	
II	4 (57.1)		
III	1 (14.3)		
IV	1 (14.3)		
2-LIFESTYLE			
Smoking status			0.2^a^
No	2 (28.6)	0 (0)	
Yes	5 (71.4)	8 (100.0)	
Regular physical activity			0.62^a^
No	4 (57.1)	3 (37.5)	
Yes	3 (42.9)	5 (62.5)	
Tap water intake			**0.04** ^*^ ^a^
No	1 (14.3)	6 (75.0)	
Yes	6 (85.7)	2 (25.0)	
Frequent antibiotics use			**0.04** ^*^ ^a^
No	2 (28.6)	7 (87.5)	
Yes	5 (71.4)	1 (12.5)	
Regular NSAID use			0.32^a^
No	3 (42.9)	6 (75.0)	
Yes	4 (57.1)	2 (25.0)	

**Notes.**

Values are presented as frequency (percentage) unless otherwise stated.

a Fisher’s Exact Test.

b independent-samples t-test.

* Bold fonts indicate statistically significant associations.

Frequent antibiotic usage and tap water consumption were significantly more prevalent among CRC patients compared to controls (*p* = 0.04 for both).

Most tumors were left-sided (85.7%), with the descending colon accounting for 57.1%, followed by the sigmoid colon and rectum, each constituting 14.3%. Stage IV disease accounted for 28.6% of total CRC cases. Abnormal bowel habits were significantly more common among CRC patients than controls (71.4% *vs.* 0%, *p* = 0.01).

Table 2 summarizes the nutrient intake among cases and controls. The control group had a significantly higher median carbohydrate intake than cases (382.3 [IQR: 247.1–482.7] *vs.* 126.3 [IQR: 100.5–254.5] g/day; *p* value = 0.005). There was no significant difference between the two groups in terms of protein, fat, and fiber intake.

**Table 2:** Daily nutrient intake of colorectal cancer patients and healthy controls.

Nutrient	CRC cases (*n* = 7)	Controls (*n* = 8)	*P* value
Carbohydrates (g/day) median (IQR)	126.3 (100.5, 254.5)	382.3 (247.1, 482.7)	0.005^*^ ^a^
Protein (g/day)	62.5 ± 33.2	38.4 ± 19.9	0.11^b^
Fat (g/day)	41.0 ± 30.1	20.9 ± 7.8	0.13^b^
Saturated fat (g/day)	14.8 ± 10.4	9.1 ± 3.2	0.21^b^
Unsaturated fat (g/day)	26.2 ± 19.9	11.8 ± 4.8	0.11^b^
Total fiber (g/day)	5.4 ± 2.9	4.3 ± 1.3	0.39^b^
Fiber soluble (g/day)	1.6 ± 0.9	1.3 ± 0.4	0.39^b^
Fiber insoluble (g/day)	3.8 ± 2.0	3.0 ± 0.9	0.39^b^

**Notes.**

Values are presented as mean ± SD unless otherwise stated.

a Mann–Whitney Test.

b independent-samples t-test.

* Bold fonts indicate statistically significant associations.

Figure 1 presents the frequency of consumption of selected food items among CRC patients and healthy controls, with a rate of 2 or more times per week. The selected food items included canned juice, instant coffee, processed meat, broasted chicken, canned fish, processed chicken, spicy food, and fast food. Although CRC patients reported higher consumption frequencies than controls across all selected food items, none of these differences was statistically significant as assessed by Fisher’s exact test.

**Figure 1:**
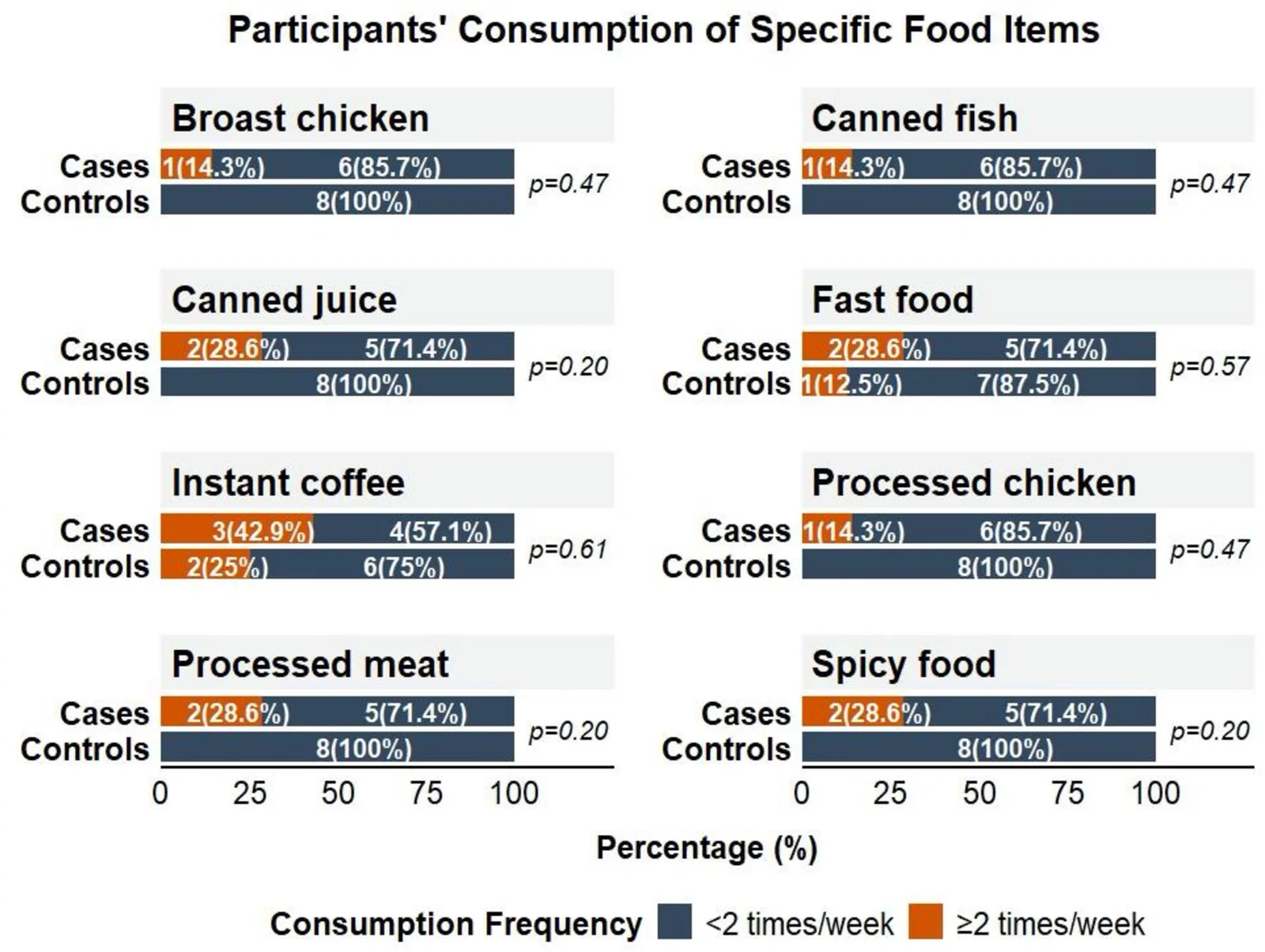
Distribution of the consumption of selected food items among cases and controls. Data expressed as n (%). The characteristics of the two groups were compared using Fisher’s exact test.

### Alpha and beta diversity

Statistical analyses revealed no significant differences in overall microbial alpha diversity between CRC patients and healthy controls. Shannon, Simpson, and observed species indices did not differ significantly between groups according to Wilcoxon rank-sum tests (Data S3).

To evaluate overall microbial community composition, beta diversity was assessed using Bray–Curtis dissimilarities and examined using PERMANOVA. Disease status explained 9.3% of variation in microbial composition (*R^2^* = 0.093), but no statistically significant separation between CRC patients and healthy controls was detected (*p* = 0.104).

Analysis of within-group dispersion demonstrated significantly greater inter-individual microbial heterogeneity among CRC participants compared with controls. Pairwise within-group Bray–Curtis distances were significantly higher in the CRC group (median = 0.882, IQR = 0.136) than in controls (median = 0.714, IQR = 0.118; Wilcoxon *p* = 0.000864), indicating greater variability in microbial community composition among CRC samples (Fig. 2).

**Figure 2:**
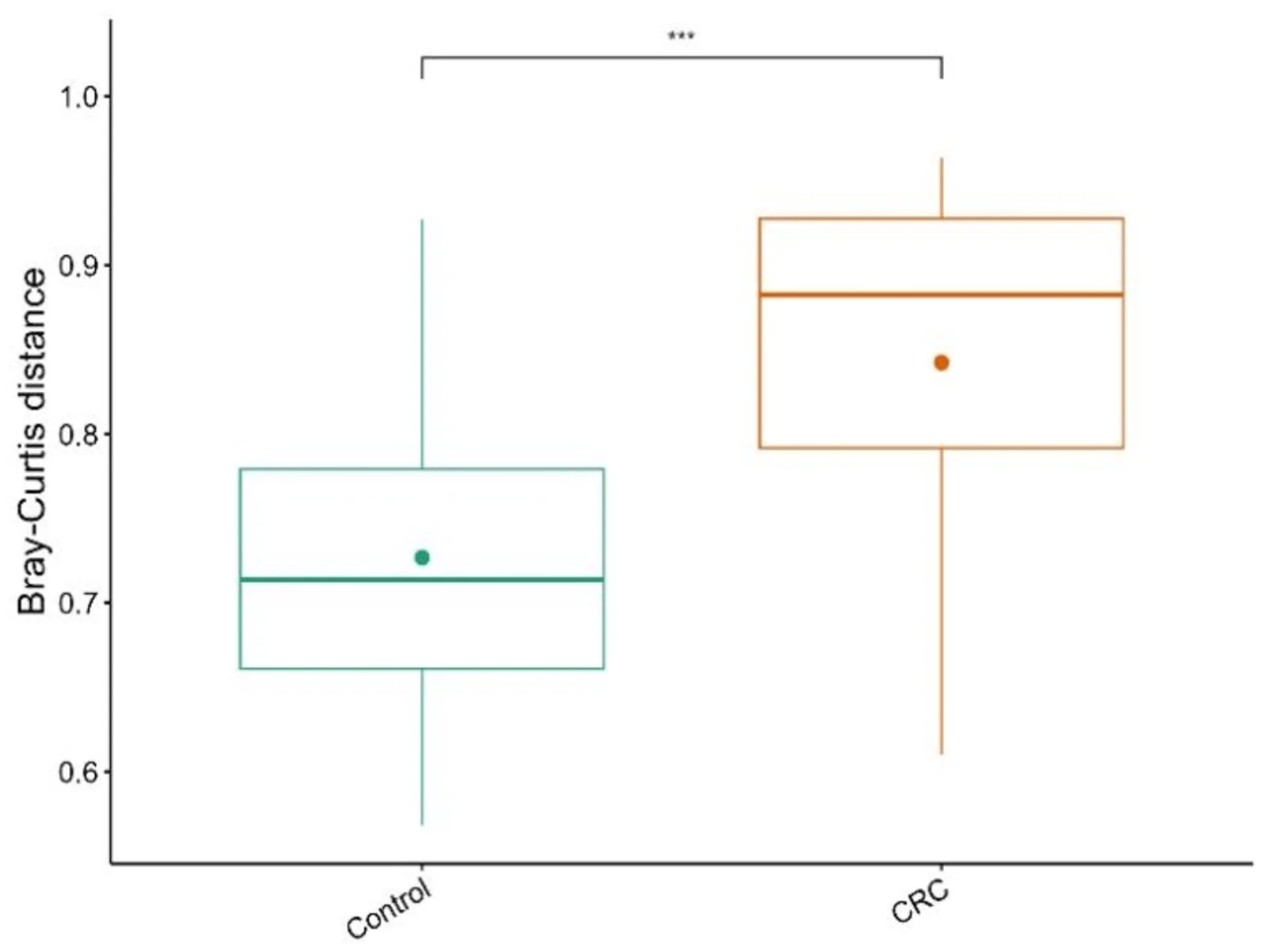
Boxplot showing the distribution of within-group pairwise Bray–Curtis dissimilarities among CRC patients and controls. The CRC group exhibited significantly greater within-group variation than the control group (*p* = 0.000864, Wilcoxon rank-sum test), indicating increased inter-individual heterogeneity in microbial community composition among CRC patients. Asterisks indicate statistical significance (*** *p* < 0.001).

To further explore factors associated with microbial community variation, multivariable PERMANOVA incorporating disease status together with selected demographic and dietary variables was performed. Processed chicken consumption was associated with beta diversity variation (*R^2^* = 0.098, *p*=* 0.038*), whereas disease status (*R^2^* = 0.071, *p* = 0.281), age (*R^2^* = 0.036, *p* = 0.973), sex (*R^2^* = 0.159, *p* = 0.125), fast-food intake (*R^2^* = 0.062, *p* = 0.469), and spicy food consumption (*R^2^* = 0.060, *p* = 0.464) were not significantly associated with microbial community composition (Data S4). Homogeneity of multivariate dispersion analysis for processed chicken consumption did not demonstrate significant differences between groups (permutation test, *p* =* 0.628*).

Additional exploratory multifactorial PERMANOVA analyses incorporating disease status together with individual dietary and lifestyle variables were also performed. Across these analyses, processed chicken consumption demonstrated the strongest association with microbial community variation (*R^2^* = 0.116, adjusted *p*=* 0.016*) (Data S5).

### Differentially abundant taxa between CRC patients and controls

Differential abundance analysis using the metagenomeSeq zero-inflated log-normal model was performed to compare genus-level microbial profiles between CRC patients and healthy controls (Data S6). Several Lachnospiraceae-related taxa demonstrated differential abundance patterns, including higher relative abundance of *Lachnospiraceae CAG-56* (logFC = −3.76, adjusted *p*=* 3.*9 ×* 10* ^−7^) and *Coprococcus_2* (logFC = −3.23, adjusted *p*=* 0.0004*) in controls, whereas *Lachnospiraceae_UCG-010* (logFC = 1.41, adjusted *p* = 0.035) was more abundant in CRC samples (Data S6).

Additional differential abundance analyses were performed within the CRC cohort to investigate associations with dietary and lifestyle variables (Data S7). For dietary and lifestyle variables, participants reporting exposure at least once per week (“Yes”) were compared with those reporting no exposure (“No”). Positive logFC values indicate a higher relative abundance in the exposed group. The number of CRC participants within each dietary subgroup is provided in Data S8.

Regular consumption of spicy food was associated with higher abundance of multiple taxa, including *Ruminococcaceae UCG-010, Hydrogenoanaerobacterium, Christensenellaceae R-7, Butyrivibrio, Intestinimonas*, and members of the *Clostridiales vadinBB60* and *Prevotellaceae* groups (all logFC > 2, FDR-adjusted *p* < 0.05) (Data S7 and Fig. 3).

**Figure 3:**
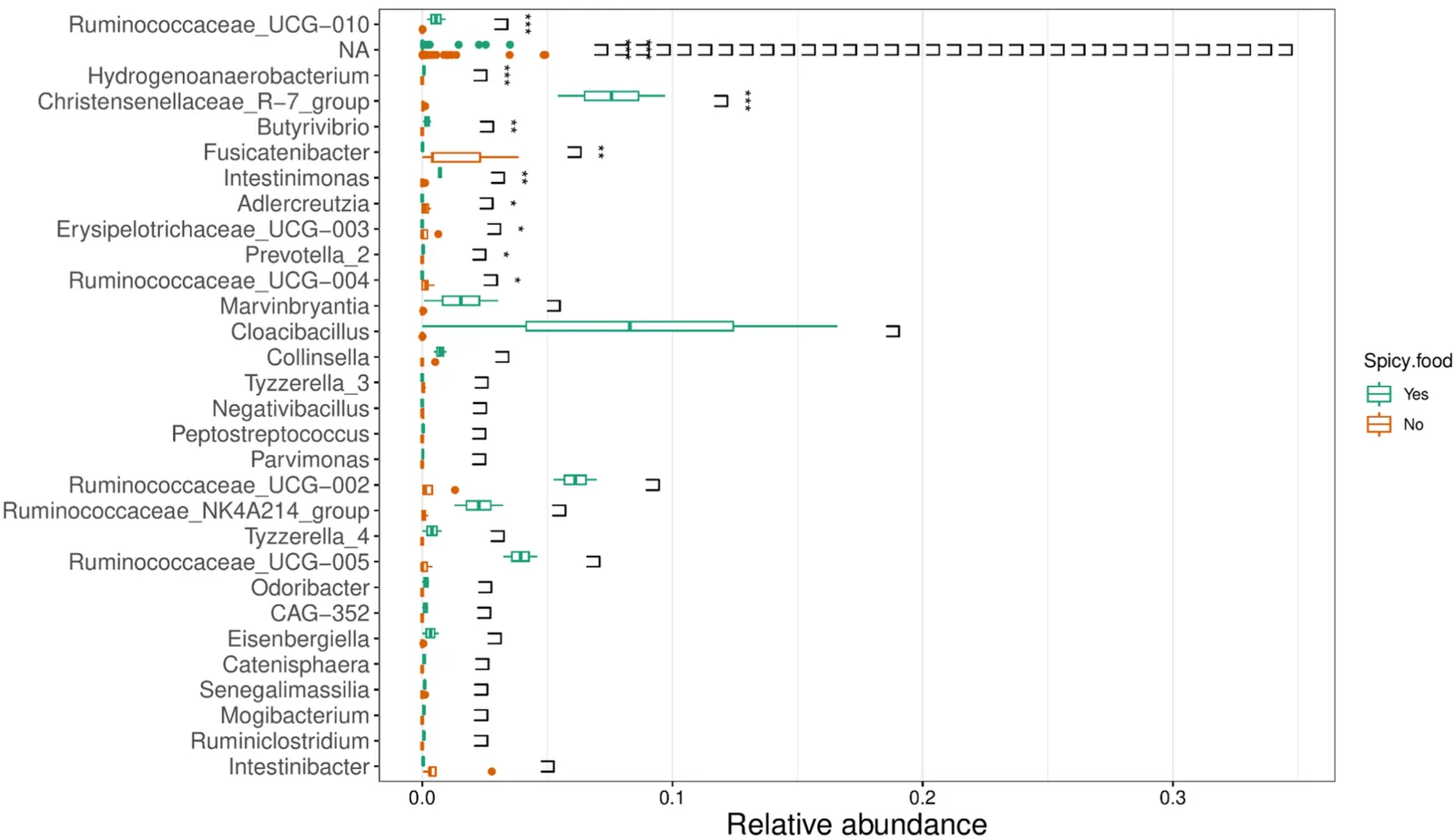
Differential abundance of microbial genera among colorectal cancer (CRC) patients based on spicy food consumption. CRC patients who consumed spicy food at least once per week (‘Yes’) *versus* those who did not (‘No’). The bar plot presents genera with significantly different abundances identified by metagenomeSeq analysis (adjusted *p*-value < 0.05); NA represents unknown genus.

Patients who reported consuming processed chicken at least once per week demonstrated differential abundance of several taxa (Data S7 and Fig. S1), including increased abundance of *Eisenbergiella, Corynebacterium_1, Corynebacterium*, and *Peptoniphilus* (all logFC > 2, FDR-adjusted *p* < 0.001).

Among patients who consumed canned juice at least once per week, *Intestinimonas* showed higher abundance (logFC = 4.57, FDR-adjusted *p* = *0.001*), whereas *Actinomyces*, *Intestinibacter,* and members of the Streptococcaceae family demonstrated lower abundance (logFC < −4.5 and FDR-adjusted *p* < 0.05) (Data S7).

Frequent fast-food consumption (≥1 time/week) was associated with increased abundance of *Akkermansia* (logFC = 6.15, FDR-adjusted *p*=* 0.02*) (Data S7).

Sex-associated differences in microbial composition were also observed within the CRC cohort. *Collinsella*, *Senegalimassilia*, *Olsenella*, *Howardella*, and *Prevotella_9* were more abundant in male participants (logFC > 2, FDR-adjusted *p* < 0.05), whereas *Adlercreutzia* was less abundant in males (logFC = −3.04, FDR-adjusted *p* = *0.04*) (Data S7).

## Discussion

CRC remains a significant worldwide health threat, with growing incidence in developing countries, including the Middle East and North Africa region (Sung et al., 2021; Siegel et al., 2022; Torre et al., 2015; Arnold et al., 2017).

In this pilot case-control Egyptian study, we detected distinct patterns of gut microbial heterogeneity, genus-level taxonomic shifts, and functional differences related to CRC, in addition to dietary patterns, physical activity, antibiotic use, water source, and sex.

Lifestyle assessment revealed that frequent antibiotic use and tap water consumption were more prevalent in the CRC patients (adjusted *p* = 0.04). Previous studies have shown that long-term consumption of chlorinated drinking water is associated with CRC (Tafesse et al., 2022), with disinfection by-products formed during water treatment also proposed as potential contributors (Helte et al., 2023). Moreover, growing evidence supports a link between drinking water quality and gut microbial composition (Moghaddam, Abkar & Fowler, 2024). Notably, the association between chlorinated tap water and CRC may be particularly pronounced in low-to-middle income countries (LMICs) such as Egypt, where reliance on heavily chlorinated municipal water and higher exposure to disinfection by-products may elevate cancer risk compared with high-income countries that employ more advanced multi-stage water treatment technologies. Antibiotic exposure has also been linked to microbial alterations and to colon tumor development across different age groups (McDowell et al., 2022; Pandey et al., 2023), highlighting the role of these environmental factors in influencing CRC microbial profiles.

At the taxonomic level, several members of the Lachnospiraceae family, such as *Lachnospiraceae CAG-56* and *Coprococcus_2,* were more abundant in healthy controls, whereas *Lachnospiraceae UCG-010* was enriched in CRC patients. This finding is consistent with previous reports linking *Lachnospiraceae UCG-010 (id.11330)* to CRC development, through different mechanisms reported to be independent of IL-10-mediated immune regulation (Ma et al., 2024).

Notably, members of the family Lachnospiraceae exhibit considerable functional heterogeneity. While certain genera in the family Lachnospiraceae have been associated with immune modulation (Zhang et al., 2023; Bullman et al., 2017; Qin et al., 2010; Kasahara et al., 2018; Bajaj et al., 2012) and the production of short-chain fatty acids, particularly butyrate (Louis & Flint, 2009; Chang et al., 2014; Arpaia et al., 2013; Koh et al., 2016; Belcheva et al., 2014), others have been linked to pro-inflammatory processes and metabolic disorders, including type 2 diabetes and non-alcoholic fatty liver disease (Wang et al., 2012; Chua et al., 2018; Shen et al., 2017). This intra-family heterogeneity highlights the importance of species- and strain-level resolution in microbiome–cancer research and suggests that functional attributes, rather than taxonomic classification alone, may be the primary determinants of biological impact (Sorbara et al., 2020).

CRC patients also exhibited greater inter-individual variability in gut microbial composition than controls, with higher pairwise within-group Bray–Curtis distances. Greater intra-tumoral microbial heterogeneity has been previously linked to adenoma–carcinoma progression and CRC molecular subtypes (*e.g.*, oncogenic mutations, microsatellite instability) (Liu et al., 2021b). However, inconsistent findings across studies, with some reporting decreased diversity in CRC patients compared to healthy controls (Park et al., 2021), likely stem from variations in sampling strategies (tissue *vs.* fecal), analytic methods, and population demographics.

In terms of dietary patterns, the control group had higher carbohydrate consumption than CRC patients (adjusted *p*=* 0.005*), whereas protein, fat, and fiber intake did not differ significantly between groups. These results differ from those of Jonoush et al. (2022), who reported a positive association between CRC and high total carbohydrate intake, yet are consistent with recent evidence (Xiao et al., 2024; Ai et al., 2019; Bouvard et al., 2015) demonstrating that carbohydrate quality, fermentability and resistant starch content may be more relevant than overall quantity in influencing CRC risk and microbial metabolic activity. Notably, the Food Questionnaire data in this study suggest that the higher carbohydrate intake observed in controls was largely driven by complex carbohydrate sources—such as cereals, legumes, and vegetables—rather than simple sugars, consistent with a more plant-based dietary pattern. This distinction is clinically relevant, as complex carbohydrates promote the growth of beneficial bacteria (*e.g.*, butyrate producers), whereas simple sugars are associated with pro-inflammatory microbial profiles.

Additional relationships were identified between specific dietary factors and microbial taxa in CRC patients. Spicy food consumption was associated with enrichment of the genera *Ruminococcaceae UCG-010, Hydrogenoanaerobacterium, Christensenellaceae R-7, Butyrivibrio, Intestinimonas,* and members of the *Clostridiales vadinBB60* and *Prevotellaceae* groups. Although data on spice-derived phytochemicals are scarce, available evidence suggests their potential to modulate gut microbial composition and metabolic activity (Dacrema et al., 2022), highlighting the need for further research at physiologically relevant exposure levels.

Processed chicken consumption was associated with a higher abundance of taxa, including *Eisenbergiella* and *Peptoniphilus.* This is consistent with earlier work showing that high intake of ultra-processed foods is associated with an increased risk of colorectal adenomas and serrated lesions (He, Cheng & Xing, 2021; Hang et al., 2023), and it is further supported by experimental evidence demonstrating that pesco-vegetarian diets may exert protective effects against colon tumorigenesis (De Filippo et al., 2024).

The increased abundance of *Peptoniphilus* observed in this study is notable, as previous reports have linked this genus to several malignancies, including prostate, breast, and oral squamous cell carcinoma (Hurst et al., 2024). Similarly, altered abundance of *Eisenbergiella* has been described in studies investigating obesity, metabolic disorders, and disturbed gut microbial ecosystems (Mao et al., 2025; Li et al., 2025). While the biological significance of these associations remains unclear, they warrant additional investigation in larger studies.

Finally, sex-associated differences in microbial composition were observed within the CRC cohort, with several genera, including *Collinsella*, *Senegalimassilia*, *Olsenella*, *Howardella*, and *Prevotella_9*, showing greater abundance among male CRC participants, whereas *Adlercreutzia* demonstrated lower abundance in males. These observations are consistent with previous evidence suggesting sex-related variation in gut microbial composition (Hawley, Forster & Giles, 2025; Wang et al., 2023). However, given the limited sample size and inability to robustly assess comparable sex-stratified patterns in the control cohort, these findings should be interpreted cautiously and considered exploratory rather than evidence of CRC-specific sex effects. Future studies with larger cohorts are required to determine whether sex-associated microbial variation contributes to differences in CRC susceptibility, progression, or treatment response.

This study provides one of the first region-specific pilot studies on gut microbiota in Egyptian patients with CRC and explores their associations with dietary and lifestyle factors. In addition, the study was conducted and reported in accordance with the Strengthening the Reporting of Observational Studies in Epidemiology (STROBE) guidelines (Von Elm et al., 2007).

A number of limitations should be acknowledged. The study had a small sample size and a single-center cross-sectional design, which may limit generalizability and statistical power. Dietary and lifestyle information was collected through interviewer-administered questionnaires and remained dependent on participant recall, making it subject to recall bias. The exploratory assessment of multiple dietary variables increased the risk of false-positive findings, and the overlap between processed meat and processed chicken consumption limited our ability to distinguish their independent effects. Further to this, CRC participants were significantly younger than controls, although multivariable analyses did not identify age as a significant contributor to overall microbial community composition. The higher proportion of female participants may also have influenced the findings and limited the generalizability of the results. Differential abundance analyses were performed at the genus level using metagenomeSeq; while genus-level aggregation reduced sparsity and improved interpretability, it may have obscured ASV-level variation. Therefore, all findings should be considered preliminary and hypothesis-generating and require validation in larger, multi-center, age-matched cohorts.

## Conclusions

This pilot case-control study provides the first comprehensive assessment of gut microbial composition, predicted functional traits, and dietary and lifestyle factors in Egyptian patients with CRC. The findings indicate increased microbial heterogeneity and distinct associations between gut microbial profiles, dietary patterns, physical activity, and sex, highlighting the potential impact of modifiable environmental factors on CRC-related microbiota profiles. Larger, multi-center studies integrating multi-omics approaches are needed to validate these findings and clarify causal relationships.

## Supplemental Information

10.7717/peerj.21829/supp-1 Supplemental Information 1 Differential abundance of microbial genera among colorectal cancer (CRC) patients based on processed chicken consumption CRC patients who either consume processed chicken more than once a week (“Yes”) versus those who do not (“No”). The bar plot presents genera with significantly different abundances identified by metagenomeSeq analysis (adjusted *p* -value < 0.05).

10.7717/peerj.21829/supp-2 Supplemental Information 2 Data

10.7717/peerj.21829/supp-3 Supplemental Information 3 Code for Raw Dataset

10.7717/peerj.21829/supp-4 Supplemental Information 4 Numerical raw data

10.7717/peerj.21829/supp-5 Supplemental Information 5 STROBE checklist

## Additional Information and Declarations

### Field Study Permissions

The following information was supplied relating to field study approvals (i.e., approving body and any reference numbers): The study was conducted at the Department of Oncology and Nuclear Medicine, Kasr Al Aini Hospital, Cairo University, Cairo, Egypt, where biological specimens and participant data were collected from colorectal cancer patients and healthy controls.

### Data Availability

The following information was supplied regarding data availability: The data is available in the Supplemental Files. The raw sequence data are available at NCBI BioProject: PRJNA1311352.

## References

[ref-1] Abotaleb A (2018). Cost of illness for colorectal cancer at low middle income countries Egypt case. Annals of Oncology.

[ref-2] Ai D, Pan H, Li X, Gao Y, Liu G, Xia L (2019). Identifying gut microbiota associated with colorectal cancer using a zero-inflated lognormal model. Frontiers in Microbiology.

[ref-3] Arnold M, Sierra MS, Laversanne M, Soerjomataram I, Jemalm A, Bray F (2017). Global patterns and trends in colorectal cancer incidence and mortality. Gut.

[ref-4] Arpaia N, Campbell C, Fan X, Dikiy S, vander Veeken J, De Roos P, Liu H, Cross JR, Pfeffer K, Coffer PJ, Rudensky AY (2013). Metabolites produced by commensal bacteria promote peripheral regulatory T-cell generation. Nature.

[ref-5] Baas FS, Brusselaers N, Nagtegaal ID, Engstrand L, Boleij A (2024). Navigating beyond associations: opportunities to establish causal relationships between the gut microbiome and colorectal carcinogenesis. Cell Host & Microbe.

[ref-6] Bajaj J, Hylemon PB, Ridlon JM, Heuman DM, Daita K, White MB, Monteith P, Noble NA, Sikaroodi M, Gillevet PM (2012). Colonic mucosal microbiome differs from stool microbiome in cirrhosis and hepatic encephalopathy and is linked to cognition and inflammation. American Journal of Physiology. Gastrointestinal and Liver Physiology.

[ref-7] Belcheva A, Irrazabal T, Robertson SJ, Streutker C, Maughan H, Rubino S, Moriyama EH, Copeland JK, Kumar S, Green B, Stintzi A, Martin A (2014). Gut microbial metabolism drives transformation of msh2-deficient colon epithelial cells. Cell.

[ref-8] Bouvard V, Loomis D, Guyton KZ, Grosse Y, El Ghissassi F, Benbrahim-Tallaa L, Guha N, Mattock H, Straif K (2015). Carcinogenicity of consumption of red and processed meat. The Lancet Oncology.

[ref-9] Bullman S, Pedamallu CS, Sicinska E, Clancy TE, Zhang X, Cai D, Neuberg D, Huang K, Guevara F, Nelson T, Chipashvili O, Hagan T, Walker M, Ramachandran A, Diosdado B, Serna G, Mulet N, Landolfi S, Ramony Cajal S, Fasani R, Aguirre AJ, Ng K, Élez E, Ogino S, Tabernero J, Fuchs CS, Hahn WC, Nuciforo P, Meyerson M (2017). Analysis of Fusobacterium persistence and antibiotic response in colorectal cancer. Science.

[ref-10] Callahan BJ, McMurdie PJ, Rosen MJ, Han AW, Johnson AJA, Holmes SP (2016). DADA2: high-resolution sample inference from Illumina amplicon data. Nature Methods.

[ref-11] Chang PV, Hao L, Offermanns S, Medzhitov R (2014). The microbial metabolite butyrate regulates intestinal macrophage function *via* histone deacetylase inhibition. Proceedings of the National Academy of Sciences of the United States of America.

[ref-12] Chua HH, Chou HC, Tung YL, Chiang BL, Liao CC, Liu HH, Ni YH (2018). Intestinal dysbiosis featuring abundance of *Ruminococcus gnavus* associates with allergic diseases in infants. Gastroenterology.

[ref-13] Dacrema M, Parodi B, Dagna L, Polito L, Masi M, Salvatore F, Daglia M (2022). Spice-derived bioactive compounds confer colorectal cancer prevention *via* modulation of gut microbiota. Cancer.

[ref-14] De Filippo C, Chioccioli S, Meriggi N, Troise AD, Vitali F, Mejia Monroy M, Özsezen S, Tortora K, Balvay A, Maudet C, Naud N, Fouché E, Buisson C, Dupuy J, Bézirard V, Chevolleau S, Tondereau V, Theodorou V, Maslo C, Aubry P, Etienne C, Giovannelli L, Longo V, Scaloni A, Cavalieri D, Bouwman J, Pierre F, Gérard P, Guéraud F, Caderni G (2024). Gut microbiota drives colon cancer risk associated with diet: a comparative analysis of meat-based and pesco-vegetarian diets. Microbiome.

[ref-15] Felsenstein J (1981). Evolutionary trees from DNA sequences: a maximum likelihood approach. Journal of Molecular Evolution.

[ref-16] Gado AS, Ebeid BA, Abdelmohsen AM, Gado TS, Axon ATR (2016). Quality of colonoscopy practice: a single-center experience in Egypt. The Egyptian Journal of Internal Medicine.

[ref-17] GBD 2019 Colorectal Cancer Collaborators (2022). Global, regional, and national burden of colorectal cancer and its risk factors, 1990–2019: a systematic analysis for the Global Burden of Disease Study 2019. The Lancet. Gastroenterology & Hepatology.

[ref-18] Ginestet C (2011). ggplot2: elegant graphics for data analysis. Journal of the Royal Statistical Society Series A.

[ref-19] Hang D, Zong X, Li X, Li N, Huang Y, Xu X, Cao Y, Wu K, Cao Y, Giovannucci EL, Song M, Wang M, Chan AT, Fang Z, Zhang FF (2023). Ultra-processed food consumption and risk of colorectal cancer precursors: results from three prospective cohorts. Journal of the National Cancer Institute.

[ref-20] Hassan A, Khalaf A, Elias A (2021). Colorectal cancer in Egypt: clinical, life-style, and socio-demographic risk factors. Al-Azhar International Medical Journal.

[ref-21] Hawley JA, Forster SC, Giles EM (2025). Exercise, the gut microbiome and gastrointestinal diseases: therapeutic impact and molecular mechanisms. Gastroenterology.

[ref-22] He T, Cheng X, Xing C (2021). The gut microbial diversity of colon cancer patients and the clinical significance. Bioengineered.

[ref-23] Helte E, Säve-Söderbergh M, Larsson SC, Martling A, Åkesson A (2023). Disinfection by-products in drinking water and risk of colorectal cancer: a population-based cohort study. Journal of the National Cancer Institute.

[ref-24] Hurst R, Brewer DS, Gihawi A, Wain J, Cooper CS (2024). Cancer invasion and anaerobic bacteria: new insights into mechanisms. Journal of Medical Microbiology.

[ref-25] Huycke MM, Abrams V, Moore DR (2002). Enterococcus faecalis produces extracellular superoxide and hydrogen peroxide that damages colonic epithelial cell DNA. Carcinogenesis.

[ref-26] Jafari A, Alamdarloo PM, Dehghani M, Bastani P, Ravangard R (2021). Economic burden of colorectal cancer: a case of fars, Iran. Cancer Control: Journal of the Moffitt Cancer Center.

[ref-27] Jonoush M, Fathi S, Hassanpour Ardekanizadeh N, Khalatbari Mohseni G, Majidi N, Keshavarz SA, Shekari S, Nemat Gorgani S, Torki SA, Sotoudeh M, Habibi F, Gholamalizadeh M, Alizadeh A, Doaei S (2022). The association between different types of dietary carbohydrates and colorectal cancer: a case–control study. Frontiers in Nutrition.

[ref-28] Kasahara K, Krautkramer KA, Org E, Romano KA, Kerby RL, Vivas EI, Mehrabian M, Denu JM, Bäckhed F, Lusis AJ (2018). Interactions between Roseburia intestinalis and diet modulate atherogenesis in a murine model. Nature Microbiology.

[ref-29] Koh A, De Vadder F, Kovatcheva-Datchary P, Bäckhed F (2016). From dietary fiber to host physiology: short-chain fatty acids as key bacterial metabolites. Cell.

[ref-30] Li G, Cheng Y, Yang X, Chai Z, Mu Z, Chao H, Li H, Qi Y, Qi L, Liu J (2025). Integrated gut microbiota and serum metabolomics reveal glyphosate-induced hepatic injury in mice. Human and Experimental Toxicology.

[ref-31] Liu C, Cui Y, Li X, Yao M (2021a). microeco: an R package for data mining in microbial community ecology. FEMS Microbiology Ecology.

[ref-32] Liu W, Zhang X, Xu H, Li S, Lau HCH, Chen Q, Zhang B, Zhao L, Chen H, Sung JJY, Yu J (2021b). Microbial community heterogeneity within colorectal neoplasia and its correlation with colorectal carcinogenesis. Gastroenterology.

[ref-33] Louis P, Flint HJ (2009). Diversity, metabolism and microbial ecology of butyrate-producing bacteria from the human large intestine. FEMS Microbiology Letters.

[ref-34] Ma M, Zheng Z, Li J, He Y, Kang W, Ye X (2024). Association between the gut microbiota, inflammatory factors, and colorectal cancer: evidence from Mendelian randomization analysis. Frontiers in Microbiology.

[ref-35] Maddocks ODK, Short AJ, Donnenberg MS, Bader S, Harrison DJ (2009). Attaching and effacing Escherichia coli downregulate DNA mismatch repair protein *in vitro* and are associated with colorectal adenocarcinomas in humans. PLOS ONE.

[ref-36] Mao X, Paerhati G, Wu Y, Cheng LF (2025). Modulation of gut microbiota, up-regulation of ZO-1, and promotion of metabolism as therapeutic mechanisms of indole-3-carbinol against obesity in mice. Frontiers in Pharmacology.

[ref-37] McDowell R, Perrott S, Murchie P, Cardwell C, Hughes C, Samuel L (2022). Oral antibiotic use and early-onset colorectal cancer: findings from a case–control study using a national clinical database. British Journal of Cancer.

[ref-38] McMurdie PJ, Holmes S (2013). phyloseq: an R package for reproducible interactive analysis and graphics of microbiome census data. PLOS ONE.

[ref-39] Moghaddam HS, Abkar L, Fowler SJ (2024). Making waves: from tap to gut—exploring the impact of drinking water on gut microbiota. Water Research.

[ref-40] National Nutrition Institute (NNI) (2006). Food composition tables for Egypt.

[ref-41] Nielsen DE, Boucher BA, Da Costa LA, Jenkins DJA, El-Sohemy A (2023). Reproducibility and validity of the Toronto-modified Harvard food frequency questionnaire in a multi-ethnic sample of young adults. European Journal of Clinical Nutrition.

[ref-42] Orangio GR (2018). The economics of colon cancer. Surgical Oncology Clinics of North America.

[ref-43] Pandey H, Tang DWT, Wong SH, Lal D (2023). Gut microbiota in colorectal cancer: biological role and therapeutic opportunities. Cancer.

[ref-44] Park J, Kim N-E, Yoon H, Shin CM, Kim N, Lee DH, Park JY, Choi CH, Kim JG, Kim Y-K, Shin T-S, Yang J, Park YS (2021). Fecal microbiota and gut microbe-derived extracellular vesicles in colorectal cancer. Frontiers in Oncology.

[ref-45] Paulson JN, Stine OC, Bravo HC, Pop M (2013). Differential abundance analysis for microbial marker-gene surveys. Nature Methods.

[ref-46] Qin J, Li R, Raes J, Arumugam M, Burgdorf KS, Manichanh C, Nielsen T, Pons N, Levenez F, Yamada T, Mende DR, Li J, Xu J, Li S, Li D, Cao J, Wang B, Liang H, Zheng H, Xie Y, Tap J, Lepage P, Bertalan M, Batto J-M, Hansen T, Le Paslier D, Linneberg A, Nielsen HB, Pelletier E, Renault P, Sicheritz-Ponten T, Turner K, Zhu H, Yu C, Li S, Jian M, Zhou Y, Li Y, Zhang X, Qin N, Yang H, Wang J, Brunak S, Doré J, Guarner F, Kristiansen K, Pedersen O, Parkhill J, Weissenbach J, Bork P, Ehrlich SD, Wang J (2010). A human gut microbial gene catalog established by metagenomic sequencing. Nature.

[ref-47] Quast C, Pruesse E, Yilmaz P, Gerken J, Schweer T, Yarza P, Peplies J, Glöckner FO (2013). The SILVA ribosomal RNA gene database project: improved data processing and web-based tools. Nucleic Acids Research.

[ref-48] Repass J, Maherali N, Owen K (2016). Reproducibility Project: cancer Biology. Registered report: fusobacterium nucleatum infection is prevalent in human colorectal carcinoma. ELife.

[ref-49] Saitou N, Nei M (1987). The neighbor-joining method: a new method for reconstructing phylogenetic trees. Molecular Biology and Evolution.

[ref-50] Schliep KP (2011). phangorn: phylogenetic analysis in R. Bioinformatics.

[ref-51] Shen F, Zheng RD, Sun XQ, Ding WJ, Wang XY, Fan JG (2017). Gut microbiota dysbiosis in patients with non-alcoholic fatty liver disease. Hepatobiliary & Pancreatic Diseases International.

[ref-52] Siegel RL, Miller KD, Fuchs HE, Jemal A (2022). Cancer statistics, 2022. CA: a Cancer Journal for Clinicians.

[ref-53] Singhal S, Bhadana R, Jain BP, Gautam A, Pandey S, Rani V (2023). Role of gut microbiota in tumorigenesis and antitumoral therapies: an updated review. Biotechnology & Genetic Engineering Reviews.

[ref-54] Sorbara MT, Littmann ER, Fontana E, Moody TU, Kohout CE, Gjonbalaj M, Eaton V, Seok R, Leiner IM, Pamer EG (2020). Functional and genomic variation between human-derived isolates of Lachnospiraceae reveals inter- and intra-species diversity. Cell Host Microbe.

[ref-55] Sung H, Ferlay J, Siegel RL, Laversanne M, Soerjomataram I, Jemal A, Bray F, Global Cancer Statistics 2020 (2021). GLOBOCAN Estimates of Incidence and Mortality Worldwide for 36 Cancers in 185 Countries. CA: A Cancer Journal for Clinicians.

[ref-56] Tafesse N, Porcelli M, Gari SR, Ambelu A (2022). Drinking water source, chlorinated water, and colorectal cancer: a matched case–control study in Ethiopia. Environmental Health Insights.

[ref-57] Tjalsma H, Boleij A, Marchesi JR, Dutilh BE (2012). A bacterial driver–passenger model for colorectal cancer: beyond the usual suspects. Nature Reviews Microbiology 2012 10:8.

[ref-58] Torre LA, Bray F, Siegel RL, Ferlay J, Lortet-Tieulent J, Jemal A (2015). Global cancer statistics, 2012. CA: A Cancer Journal for Clinicians.

[ref-59] Von Elm E, Altman DG, Egger M, Pocock SJ, Gøtzsche PC, Vandenbroucke JP, STROBE Initiative (2007). The strengthening the reporting of observational studies in epidemiology (STROBE) statement: guidelines for reporting observational studies. PLOS Medicine.

[ref-60] Wang J, Qin J, Li Y, Cai Z, Li S, Zhu J, Zhang F, Liang S, Zhang W, Guan Y, Shen D, Peng Y, Zhang D, Jie Z, Wu W, Qin Y, Xue W, Li J, Zeng B, Li L, Wu J, Xu J, Li R, Yuan Z, Li D, Ji B, Liang X, Zheng H, Xie Y, Li S, Hu Y, Piceno YM, De Santis TZ, Zhao F, Chen W, Zhang X, Xu X, Phelps E, Yang H, Wang J, Liu L, Doré J, Ehrlich SD, Jia H (2012). A metagenome-wide association study of gut microbiota in type 2 diabetes. Nature.

[ref-61] Wang L, Tu Y-X, Chen L, Zhang Y, Pan X-L, Yang S-Q, Zhang S-J, Li S-H, Yu K-C, Song S, Xu H-L, Yin Z-C, Yue J-Q, Ni Q-L, Tang T, Zhang J-L, Guo M, Zhang S, Yao F, Liang X-J, Chen Z-X (2023). Male-biased gut microbiome and metabolites aggravate colorectal cancer development. Advanced Science.

[ref-62] Wright ES (2016). Using DECIPHER v2.0 to analyze big biological sequence data in R. The R Journal.

[ref-63] Xiao Y, Xiang L, Jiang Y, Tang Y, Gu H, Wang Y, Peng L (2024). Carbohydrate quality, not quantity, linked to reduced colorectal cancer incidence and mortality in US populations: evidence from a prospective study. BMC Medicine.

[ref-64] Yu Y-N, Fang J-Y (2015). Gut Microbiota and Colorectal Cancer. Gastrointestinal Tumors.

[ref-65] Zhang X, Yu D, Wu D, Gao X, Shao F, Zhao M, Wang J, Ma J, Wang W, Qin X, Chen Y, Xia P, Wang S (2023). Tissue-resident Lachnospiraceae family bacteria protect against colorectal carcinogenesis by promoting tumor immune surveillance. Cell Host & Microbe.

